# Alcohol abuse may increase the risk of autoimmune connective tissue disease: a nationwide population-based cohort study

**DOI:** 10.3389/fpsyt.2023.1308245

**Published:** 2024-06-01

**Authors:** Chi-Chen Chang, Chia-Ti Wang, Hong-Mo Shih, Chung-Han Ho, Chien-Chin Hsu, Hung-Jung Lin, Yen-Wei Chiu, Chien-Cheng Huang

**Affiliations:** ^1^Department of Emergency Medicine, Chi Mei Medical Center, Tainan, Taiwan; ^2^Department of Emergency Medicine, China Medical University Hospital, Taichung, Taiwan; ^3^School of Medicine, College of Medicine, China Medical University, Taichung, Taiwan; ^4^Department of Public Health, China Medical University, Taichung, Taiwan; ^5^Department of Medical Research, Chi Mei Medical Center, Tainan, Taiwan; ^6^Department of Information Management, Southern Taiwan University of Science and Technology, Tainan, Taiwan; ^7^School of Medicine, College of Medicine, National Sun Yat-sen University, Kaohsiung, Taiwan; ^8^Department of Emergency Medicine, Taipei Medical University, Taipei, Taiwan; ^9^Department of Emergency Medicine, Kaohsiung Medical University, Kaohsiung, Taiwan; ^10^Department of Environmental and Occupational Health, College of Medicine, National Cheng Kung University, Tainan, Taiwan

**Keywords:** alcohol abuse, Asian, autoimmune connective tissue disease, male, Taiwan

## Abstract

**Objectives:**

Altered immune and inflammatory responses resulting from alcohol abuse have been implicated in increasing the risk of autoimmune connective tissue disease (ACTD). However, limited research has been conducted on this topic in the Asian population. Therefore, this study was undertaken to investigate and address this knowledge gap.

**Methods:**

Using data from Taiwan’s National Health Insurance Research Database, we identified all patients with alcohol abuse between 2000 and 2017. We selected a comparison cohort without alcohol abuse, matching them in terms of age, sex, and index date at a 3:1 ratio. We collected information on common underlying comorbidities for analysis. Both cohorts were followed up until the diagnosis of ACTD or the end of 2018.

**Results:**

A total of 57,154 patients with alcohol abuse and 171,462 patients without alcohol abuse were included in the study. The age and sex distributions were similar in both cohorts, with men accounting for 89.8% of the total. After adjusting for underlying comorbidities, patients with alcohol abuse had a higher risk of developing ACTD [adjusted hazard ratio (AHR): 1.12, 95% confidence interval (CI): 1.01–1.25]. The stratified analysis revealed that this increased risk was specific to the male population. Additionally, besides alcohol abuse, liver disease, renal disease, coronary artery disease, and chronic obstructive pulmonary disease were identified as independent predictors for ACTD.

**Conclusion:**

This study demonstrates that alcohol abuse increases the risk of developing ACTD in the Asian population, particularly among men. Therefore, it is important to implement alcohol cessation, especially in individuals with liver disease, renal disease, coronary artery disease, and chronic obstructive pulmonary disease.

## 1 Introduction

Autoimmune connective tissue diseases (ACTDs) encompass a diverse group of chronic inflammatory conditions affecting various organs (1). These diseases exhibit global prevalence and can affect individuals of all races (2). Incidence rates vary, with a higher occurrence observed during adolescence and the 20s (2). Mixed connective tissue disease, specific subsets of ACTDs, have variable annual occurrence rates in different countries, typically ranging from 0.21 to 1.9 cases per 100,000 adults (2). ACTDs demonstrate sex disparities, primarily affecting women (2). Reported sex ratios vary significantly, ranging from 3.3:1 up to 16:1 (2). ACTDs have a multifactorial etiology involving genetic, environmental, and immunological factors (3). Alcohol consumption has been suggested as a potential contributing factor to the development of ACTDs (3).

Alcohol abuse is a significant public health problem worldwide, with an estimated 3.3 million deaths attributable to its harmful use in 2016 alone (4). In addition to its well-known effects on liver, cardiovascular, and neurological systems, alcohol abuse has been linked to immune dysfunction, including alterations in cytokine profiles and impaired immune cell function (5, 6). These immunomodulatory effects of alcohol have raised concerns about its possible association with autoimmune disorders.

While there is an increasing body of literature indicating a potential association between alcohol consumption and autoimmune disorders, including ACTDs, the available evidence remains inconclusive, with a lack of studies focusing on this issue in the Asian population. To address this gap, we conducted a nationwide population-based cohort study to examine the potential risk of ACTDs among individuals with alcohol abuse. Our study aims to enhance the current knowledge regarding the potential link between alcohol abuse and ACTDs, as well as shed light on the underlying mechanisms that may contribute to this association.

## 2 Methods

### 2.1 Data source

We collected data from the National Health Insurance Research Database (NHIRD), derived from Taiwan’s single-payer compulsory National Health Insurance program, covering up to 99% of the 23 million Taiwanese population (7). The NHIRD contains information on the dates of inpatient and outpatient visits, medical diagnosis, expenditure, and prescription details (7). All entries are linked by a unique personal identifier number; hence, the medical utilization and registration records could be obtained for each enrollee (7).

### 2.2 Study design, setting, and participants

**Figure 1:**
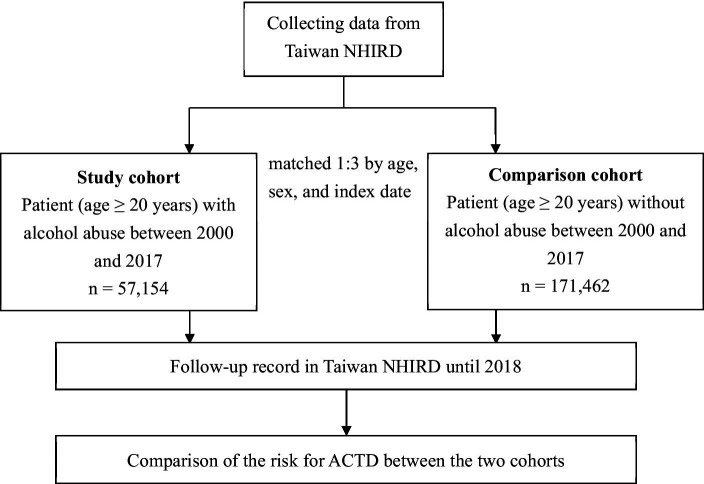
Flowchart of this study. NHIRD, National Health Insurance Research Database; ACTD, autoimmune connective tissue disease.

Alcohol abuse is recognized as a disease in Taiwan when it leads to changes in the brain and contributes to significant physical and mental health issues (8). These may include conditions such as liver disease, cardiovascular problems, cognitive impairment, and psychiatric disorders (8). The seriousness of alcohol abuse cases seeking help in hospitals in Taiwan, as in any other region, can vary widely depending on factors such as the severity of the individual’s alcohol use disorder, the presence of co-occurring health issues, and the overall impact on the person’s life (8). In our study, alcohol abuse was operationalized according to the diagnostic codes outlined by the International Classification of Diseases, Ninth Revision, Clinical Modification [ICD-9-CM] (291, 305.0, 303.0, 303.9, 357.5, 425.5, 535.3, 571.0–571.3, 655.4, or 760.71) or ICD-10 (F10.3–F10.9, F10.0, F10.1, F10.2, G62.1, G31.2, G72.1, I42.6, K29.2, K70–K70.4, K70.9, Q86.0, P04.3, O35.4, or K86.0). A patient was diagnosed as an alcohol abuser if they were above the age of 20 years and had received at least one diagnosis from the specified ICD codes during a hospitalization. The study cohort comprised all patients above the age of 20 years who met the criteria for alcohol abuse (Figure 1). The index date was defined as the date when the patients were diagnosed as alcohol abusers. Through the index date, we then identified patients without alcohol abuse as comparison cohort by matching age and sex at a ratio of 1:3. Patients who had ACTD (ICD-9-CM 710, 714 or ICD-10 M30–M36) in both cohorts before the index date were excluded.

### 2.3 Definitions of variables

We classified the collected patients into four subgroups as follows: aged 20–34, 35–49, 50–64, and ≥65 years (9). The underlying comorbidities were identified as follows: smoking (ICD-9-CM 305.1; ICD-10 Z72.0), diabetes (ICD-9-CM: 250; ICD-10 E08–E13), herpes zoster (ICD-9-CM: 053; ICD-10: B02), hepatitis (ICD-9-CM: 070; ICD-10: B15–B19), HIV infection (ICD-9-CM: 042, 079.53; ICD-10: B20–B24), liver disease (ICD-9-CM: 570–576; ICD-10: K70–K77), renal disease (ICD-9-CM: 580–593; ICD-10: N17–N19), malignancy (ICD-9-CM: 140–208; ICD-10: C00–C96, D00–D09), hypertension (ICD-9-CM: 401–405; ICD-10: I10–I16), hyperlipidemia (ICD-9-CM: 272; ICD-10: E78.5), coronary artery disease (CAD; ICD-9-CM: 410–414; ICD-10: I20–I25), congestive heart failure (CHF; ICD-9-CM: 428; ICD-10: I50), and chronic obstructive pulmonary disease (COPD; ICD-9-CM: 496; ICD-10: J44). The underlying comorbidities were defined as being diagnosed at least during one hospitalization or at three outpatient visits.

### 2.4 Outcome measures

The primary outcome measure was defined as ACTD development during the follow-up period. ACTD was identified by using the ICD-9-CM of 710 or 714 or ICD-10-CM of M30–M36 with at least one hospitalization. All patients were followed from the index date until the development of the ACTD, death, or until 31 December 2018.

### 2.5 Ethical statements

The study was conducted in accordance with the World Medical Association Declaration of Helsinki. This study was approved by the Institutional Review Board of the Chi-Mei Medical Center. As the data collected from NHIRD have been de-identified, the need for informed consent was waived.

### 2.6 Statistical analyses

We used the Pearson chi-square test and independent *t*-test to analyze categorical and continuous variables between the two cohorts. Considering the time until events occur in a cohort study, univariate and multivariate Cox proportional hazards regression analyses were performed to estimate the risk of ACTD between the alcohol abuse cohort and comparison cohort. Stratified analyses were performed according to age, sex, and underlying comorbidities to investigate potential effect modification. We also performed Cox proportional hazard regression analyses in all patients to investigate independent predictors for ACTD. All statistical analyses were performed using SAS 9.4 for Windows (SAS Institute, Cary, NC, United States). A value of *p* of <0.05 indicated significance (two-tailed).

## 3 Results

### 3.1 Patient characteristics

In this study, a total of 57,154 patients with alcohol abuse and 171,462 patients without alcohol abuse were included (Table 1). There were no significant differences between these two cohorts in terms of age and sex distributions. The largest proportion of patients belonged to the age subgroup of 35–49 years (43.2%), followed by 50–64 years (34.6%), 20–34 years (11.7%), and ≥ 65 years (10.5%). In both cohorts, male patients constituted the majority, accounting for 89.8% of the total population. Comparing the two cohorts, patients with alcohol abuse exhibited a higher prevalence of smoking, hepatitis, and liver disease. Conversely, they had a lower prevalence of conditions such as herpes zoster, HIV infection, renal disease, malignancy, CAD, and COPD than those without alcohol abuse.

**Table 1:** Comparison of demographic characteristics and underlying comorbidities between alcohol abuse and non-alcohol abuse cohorts through univariate analysis.

Variables	Alcohol abuse *n* = 57,154	Non-alcohol abuse *n* = 171,462	Value of *p*
**Age (years)**			
20–34	6,661 (11.7)	19,983 (11.7)	>0.999
35–49	24,674 (43.2)	74,022 (43.2)	
50–64	19,795 (34.6)	59,385 (34.6)	
≥65	6,024 (10.5)	18,072 (10.5)	
**Sex**			
Female	5,813 (10.2)	17,439 (10.2)	>0.999
Male	51,341 (89.8)	154,023 (89.8)	
**Underlying comorbidity**			
Smoking	367 (0.6)	941 (0.6)	0.010
Diabetes	6,729 (11.8)	20,187 (11.8)	>0.999
Herpes zoster	166 (0.3)	958 (0.6)	<0.001
Hepatitis	3,805 (6.7)	6,855 (4.0)	<0.001
HIV infection	85 (0.2)	535 (0.3)	<0.001
Liver disease	14,061 (24.6)	14,606 (8.5)	<0.001
Renal disease	2,948 (5.2)	14,151 (8.3)	<0.001
Malignancy	3,166 (5.5)	13,705 (8.0)	<0.001
Hypertension	10,269 (18.0)	30,807 (18.0)	>0.999
Hyperlipidemia	4,105 (7.2)	12,315 (7.2)	>0.999
CAD	1,862 (3.3)	9,526 (5.6)	<0.001
CHF	930 (1.6)	2,759 (1.6)	0.766
COPD	562 (1.0)	2,213 (1.3)	<0.001

Data are expressed as means ± SD or *n* (%). SD, standard deviation; HIV, human immunodeficiency virus; CAD, coronary artery disease; CHF, congestive heart failure; COPD, chronic obstructive pulmonary disease; ACTD, autoimmune connective tissue disease.

### 3.2 Comparison of two cohorts: overall analysis and stratified analyses by age, sex, and underlying comorbidity

In the overall analysis, patients with alcohol abuse exhibited a higher risk of developing ACTD than those without alcohol abuse, even after adjusting for variables that showed a significant difference in the crude analysis [adjusted hazard ratio (AHR): 1.12; 95% confidence interval (CI): 1.01–1.25, *p* = 0.037; Table 2]. Stratified analyses further revealed that the increased risk of ACTD associated with alcohol abuse was specifically observed in the male population (AHR: 1.17; 95% CI: 1.03–1.32, *p* = 0.016). However, no significant difference was found in other subgroups, including women and individuals with various underlying comorbidities included in this study.

**Table 2:** Comparison of ACTD risk between alcohol abuse and non-alcohol abuse cohorts: Cox proportional hazard regression analysis and stratified analyses by age, sex, and underlying comorbidity.

Variables	Alcohol abuse		Non-alcohol abuse		Crude HR (95% CI)	Value of *p*	AHR (95% CI)^*^	Value of *p*
	Patients, *n*	ACTD, *n* (%)	Patients, *n*	ACTD, *n* (%)				
Overall analysis	57,154	506 (0.9)	171,462	1,538 (0.9)	1.14 (1.03–1.26)	0.012	1.12 (1.01–1.25)	0.037
**Stratified analysis**								
**Age (years)**								
20–34	6,661	37 (0. 6)	19,983	121 (0.6)	1.10 (0.76–1.61)	0.611	1.02 (0.69–1.51)	0.910
35–49	24,674	205 (0.8)	74,022	569 (0.8)	1.23 (1.04–1.45)	0.014	1.19 (1.00–1.41)	0.052
50–64	19,795	202 (1.0)	59,385	633 (1.1)	1.14 (0.97–1.35)	0.114	1.11 (0.94–1.32)	0.234
≥65	6,024	62 (1.0)	18,072	215 (1.2)	1.00 (0.74–1.35)	0.990	1.05 (0.76–1.45)	0.771
**Sex**								
Female	5,813	100 (1.7)	17,439	333 (1.9)	1.05 (0.84–1.32)	0.670	0.98 (0.77–1.25)	0.886
Male	51,341	406 (0.8)	154,023	1,205 (0.8)	1.18 (1.05–1.33)	0.005	1.16 (1.03–1.31)	0.016
**Underlying comorbidity**								
Smoking	367	6 (1.6)	941	9 (1.0)	–		–	
Diabetes	6,729	65 (1.0)	20,187	191 (1.0)	1.29 (0.95–1.74)	0.102	1.27 (0.93–1.74)	0.138
Herpes zoster	166	3 (1.8)	958	10 (1.0)	–		–	
Hepatitis	3,805	42 (1.1)	6,855	66 (1.0)	–		–	
HIV infection	85	0 (0.0)	535	4 (0.8)	–		–	
Liver disease	14,061	126 (0.9)	14,606	177 (1.2)	1.07 (0.67–1.72)	0.778	1.21 (0.73–2.00)	0.464
Renal disease	2,948	24 (0.8)	14,151	158 (1.1)	0.91 (0.33–2.53)	0.863	0.87 (0.27–2.84)	0.819
Malignancy	3,166	28 (0.9)	13,705	109 (0.8)	4.70 (0.52–42.78)	0.170	4.14 (0.17–103.37)	0.387
Hypertension	10,269	127 (1.2)	30,807	344 (1.1)	1.26 (1.02–1.56)	0.035	1.18 (0.94–1.48)	0.165
Hyperlipidemia	4,105	54 (1.3)	12,315	132 (1.1)	1.36 (0.98–1.88)	0.066	1.31 (0.92–1.86)	0.130
CAD	1,862	25 (1.3)	9,526	130 (1.4)	1.05 (0.45–2.46)	0.913	0.70 (0.22–2.18)	0.533
CHF	930	11 (1.2)	2,759	14 (0.5)	–		–	
COPD	562	9 (1.6)	2,213	34 (1.5)	–		–	

ATCD, autoimmune connective tissue disease; HR, hazard ratio; CI, confidence interval; HIV, human immunodeficiency virus; AHR, adjusted hazard ratio; CAD, coronary artery disease; CHF, congestive heart failure; COPD, chronic obstructive pulmonary disease. ^*^Adjusted for variables with a significant difference in the crude analysis.

### 3.3 Independent predictors of ACTD in all participants

In addition to alcohol abuse, other factors independently associated with an increased risk of ACTD included liver disease (AHR: 1.32; 95% CI: 1.12–1.56, *p* = 0.001), renal disease (AHR: 1.21; 95% CI: 1.00–1.47), CAD (AHR: 1.30; 95% CI: 1.04–1.62, *p* = 0.020), and COPD (AHR: 1.64; 95% CI: 1.08–2.49, *p* = 0.022; Table 3).

**Table 3:** Independent predictors of ACTD in all participants by a Cox proportional hazard regression analysis.

Variables	Patients, *n*	ACTD, *n* (%)	Crude HR (95% CI)	Value of *p*	AHR (95% CI)*	Value of *p*
**Cohort**						
Alcohol abuse	57,154	506 (0.9)	1.14 (1.03–1.26)	0.012	1.12 (1.01–1.25).	0.037
Non-alcohol abuse	171,462	1,538 (0.9)	1.00 (reference)		1.00 (reference)	
**Underlying comorbidity**						
Smoking	1,308	15 (1.2)	1.37 (0.83–2.28)	0.221	–	
Herpes zoster	1,124	13 (1.2)	1.49 (0.86–2.56)	0.154	0.89 (0.46–1.71)	0.717
Hepatitis	10,660	108 (1.0)	1.46 (1.20–1.77)	<0.001	1.20 (0.93–1.55)	0.165
HIV infection	620	4 (0.7)	0.79 (0.30–2.11)	0.643	-	
Liver disease	28,667	303 (1.1)	1.53 (1.35–1.73)	<0.001	1.32 (1.12–1.56)	0.001
Renal disease	17,099	182 (1.1)	1.35 (1.16–1.57)	<0.001	1.21 (1.00–1.47)	0.050
Malignancy	16,871	137 (0.8)	1.47 (1.23–1.75)	<0.001	1.12 (0.90–1.40)	0.297
CAD	11,388	155 (1.4)	1.68 (1.43–1.98)	<0.001	1.30 (1.04–1.62)	0.020
CHF	3,689	25 (0.7)	1.01 (0.68–1.50)	0.950	–	
COPD	2,775	43 (1.6)	2.40 (1.78–3.25)	<0.001	1.64 (1.08–2.49)	0.022

ATCD, autoimmune connective tissue disease; HR, hazard ratio; CI, confidence interval; HIV, human immunodeficiency virus; AHR, adjusted hazard ratio; CAD, coronary artery disease; CHF, congestive heart failure; COPD, chronic obstructive pulmonary disease. ^*^Adjusted for variables with a significant difference in the crude analysis.

## 4 Discussion

This study demonstrated that patients with alcohol abuse had an increased risk of developing ACTD than those without alcohol abuse, particularly among men. Furthermore, apart from alcohol abuse, liver disease, renal disease, CAD, and COPD were identified as independent predictors for ACTD.

Notably, the initial alcohol abuse cohort highlighted a decreased prevalence of diseases, including herpes zoster, HIV infection, renal disease, malignancy, CAD, and COPD, when compared to individuals without alcohol abuse (Table 1). Reviewing the previous literature utilizing NHIRD, Chen and colleagues enrolled individuals with alcohol use disorder to investigate the risk of mesenteric ischemia (10). Within the alcohol use disorder cohort, there was a lower prevalence of hyperlipidemia, stroke, ischemic heart disease, congestive heart failure, peripheral artery disease, and COPD than in the non-alcohol use disorder cohort (10). Alcohol consumption may impact the adherence to medications for some chronic diseases (11). In our study, a more rigorous definition was employed (hospitalization once or outpatient visits three times with ICD codes). Consequently, the lack of follow-up in outpatient tracking may potentially lead to an underestimation of the prevalence of certain diseases.

The observed findings can be reasonably explained by the dose-dependent effects of alcohol on ACTD. While some studies have shown an increased risk of autoimmune diseases associated with alcohol consumption, others have suggested a protective effect, particularly with low-dose intake (12, 13). A recent study conducted in Denmark reported a higher risk of developing ACTD in patients with alcoholic liver cirrhosis [adjusted incidence rate ratio (aIRR): 1.84; 95% CI: 1.60–2.12] (14). Interestingly, evidence suggests that alcohol may have protective effects against various autoimmune diseases, such as autoimmune thyroid disease, systemic lupus erythematosus, rheumatoid arthritis, and multiple sclerosis, as observed in both human and animal studies (12, 15–17). However, it is important to note that alcohol exhibits pleiotropic, tissue-specific, and sex-specific anti-inflammatory actions at different doses (3). Alcohol can modulate the adaptive immune system in a dose-dependent manner, with chronic moderate consumption leading to T- and B-cell activation and proliferation and chronic heavy consumption associated with T- and B-cell depletion, apoptosis, and increased immunoglobulins (5, 18). Furthermore, excessive alcohol consumption can disrupt the gut barrier, leading to dysbiosis—an imbalance in gut microbial communities—which allows the passage of toxins, antigens, and bacteria from the gut lumen into the bloodstream (19). This process has the potential to trigger the initiation and progression of autoimmune diseases (20).

Sex- and gender-related differences exist in alcohol consumption (21). Findings from multiple countries highlight that men exhibit higher alcohol intake, more frequent drinking habits, and an increased propensity for hazardous drinking than women (21). For example, in Taiwan, the prevalence of frequent drinking is approximately six times higher among men (15.1%) than women (2.6%) (22). Our study revealed an interesting finding that the association between alcohol abuse and increased risk of ACTD was observed specifically in men, while no such association was found in women. This novel observation can be attributed to sex differences in immune response, organ vulnerability, pregnancy, sex hormones, genetic predisposition, parental inheritance, and epigenetics (23). Androgens, for instance, have been associated with decreased immune reactivity and increased threshold for the development of autoimmunity (5). It has been reported that alcohol abuse can decrease testosterone levels (24), which may contribute to the increased risk of ACTD in men. Additionally, men may have fewer vulnerability factors for ACTD than women, making alcohol abuse a significant contributing factor in their case. However, previous studies have indicated that women exhibit greater sensitivity to the effects of alcohol on inflammatory and immune responses than men (5). This difference could be attributed to variations in physiological processing, metabolic clearance of alcohol, and the sensitivity of the nervous system to alcohol (5). The topic of sex differences in relation to alcohol and its impact on ACTD is complex and requires further investigation.

The present study has notable strengths, including its nationwide design and large sample size. However, there are certain limitations that should be acknowledged. First, detailed information regarding alcohol abuse, including the quantity of alcohol consumed, was not available in the NHIRD. This lack of information may introduce confounding factors that could potentially affect the interpretation of the study results. Second, another inherent limitation of utilizing NHIRD lies in the challenge of obtaining socioeconomic status. Despite their investigation relying on insured classification and premium, the precision of these factors in representing income status lacks a solid foundation (25). Third, although an association between alcohol abuse and ATCD was identified, the causal relationship between these factors could not be definitively established due to the complex interaction between them and other comorbidities. Fourth, it should be noted that the present findings may not be generalizable to other nations due to differences in race, culture, and medical insurance systems, as well as variations in the impact of alcohol-related genetic factors among different ethnic groups. Fifth, the observed smoking rate among patients with alcohol abuse was low (0.6%), potentially influenced by the stringent diagnostic criteria. Smoking was defined as a diagnosis recorded during at least one hospitalization or three outpatient visits with specific ICD-9-CM (305.1) or ICD-10 (Z72.0) codes. Additionally, the chosen ICD codes often primarily identified patients seeking smoking cessation support. Consequently, these data might underestimate the true prevalence of smoking, indicating a non-differential misclassification bias in both alcohol abuse and non-alcohol abuse cohorts. However, this limitation is not anticipated to impact the outcomes of the study. Therefore, further studies conducted in different nations are warranted to validate these findings.

## 5 Conclusion

This population-based cohort study conducted in Taiwan revealed a noteworthy association between alcohol abuse and an increased risk of ACTD in our Asian population, with a particularly strong effect observed among men. The underlying mechanism for this association may be attributed to the dose-dependent impact of alcohol on the immune system. These findings underscore the significance of implementing focused alcohol cessation programs, public health interventions, and lifestyle modifications to prevent the onset of ACTD, especially in individuals with pre-existing conditions such as liver disease, renal disease, CAD, and COPD. However, the precise reason why alcohol abuse appears to affect men more than women in this context remains unclear. Further investigations that include detailed data on alcohol consumption levels, validation in diverse populations, and exploration of sex differences are warranted to validate and expand upon these findings.

## Data availability statement

The data used in this study were obtained from the NHIRD published by the Taiwan National Health Insurance Bureau. As per the legal restrictions outlined in the “Personal Information Protection Act” imposed by the government of Taiwan, the data cannot be publicly shared. Researchers interested in accessing the data can submit a formal proposal to the NHIRD through their website at: http://nhird.nhri.org.tw.

## Ethics statement

This study was conducted in accordance with the principles of the Declaration of Helsinki and received approval from the Institutional Review Board of the Chi Mei Medical Center. Written informed consent for participation was not required from the participants or the participants’ legal guardians/next of kin in accordance with the national legislation and institutional requirements.

## Author contributions

C-CC: Conceptualization, Writing – review & editing. C-TW: Writing – review & editing. C-HH: Writing – review & editing. C-CHs: Writing – review & editing. H-JL: Writing – review & editing. Y-WC: Writing – original draft. C-CHu: Writing – original draft. H-MS: Writing – review & editing.
